# Facts and Observations Relative to an Erysipelas Infesting the Ship's Company of the Jalouse

**Published:** 1814-06

**Authors:** George Hume Weatherhead

**Affiliations:** Surgeon to H. M. S. Jalouse


THE
### Medical and Physical Journal.

6 OF VOL. XXXI.] JUNE, 1814. [NO. 184.

"For many fortunate discoveries in medicine, and for the detection of numerous errors, the world is indebted to the rapid circulation of Monthly "Journals; and there never existed any work to which the faculty in "Europe and America were under deeper obligations than to the "Medical and Physical Journal of London, now forming a long, but an "invaluable series."—Rush.

*For the Medical and Physical Journal.*

### *Facts and Observations relative to an Erysipelas infesting the Ship's Company of the Jalouse; by* George Hume Weatherhead, Surgeon to H. M. S. Jalouse.

On joining the Jalouse in May 1813, my attention was soon put on the alert by the frequent occurrence of Erysipelas as a disease in the sick list, which at length, from becoming so prevalent, suggested the possibility that it might be contagious. Certainly no idea so readily occurred to explain a circumstance that appeared to me so singular, and I was urged by novelty sedulously to watch its propagation, and to trace if possible the history of those cases that had already occurred. The result of my inquiries did not forbid the prosecution of them, nor lessened the hope I had at first entertained of establishing a fact not generally admitted—that erysipelas is contagious.

I may observe that I had one advantage, in the present instance, peculiar to my situation. Where erysipelas occurs in a private family, even though two or three may successively have the complaint, this might never suggest the idea of infection: the superior ventilation of an house over that of a small ship; the little intercourse that may be between contiguous houses; or if even a visitor or neighbour should catch the disease, the chance that the same practitioner shall attend both cases; these taken into consideration would account for the oversight of a circumstance curious in itself, though perhaps not remarkably important as a physical consideration. In the present case, the opportunities a ship affords of making, and of ascertaining the truth of, such a remark, are more favorable. Infection in a small ship spreads rapidly, and although the contagion be weak, yet in a ship it must be conspicuous. The respiration of a number of men so crowded together soon contaminates the volume of atmosphere below, the confined vent permits but very slowly a fresh succession of pure air, the unavoidable damp between decks embodies, and therefore fixes, the contagion of effluvia, and the necessary close intercourse of confinement propagates infection by contact. On the justness and reality of these circumstances, I trust the succeeding inferences are neither faithless nor unfounded.

To put the inferences at issue in a fair point of view, I subjoin a list of the men affected, copied from the sick list on-board, with the date of the attack and disappearance of the disease as put on or discharged the sick list, with concise remarks as to the part affected and contiguity of situation connecting the chain of infection from one birth to another.

| Men's Names. | When put on sick list. | When disch[d] sick list. | Part affected, and Remarks on the Cases. |
|---|---|---|---|
| Tho. Wilson | May 12 | May 24 | The disease first appeared as a pustule two inches below the knee, surrounded with an erysipelatous areola, which extended considerably in a few days—took two doses of a neutral salt, and had a bandage, kept constantly wetted with rum, applied round the part affected, until the disease disappeared. He is the next messmate of Philips, Grossard, and Cassey, persons who afterwards had the erysipelas. |
| Thomas Smith | — 25 | — 31 | A similar case in every respect, and received the same treatment. He is next messmate to Rozer and John Smith, persons afterwards affected. |
| Patrick Kane | — 25 | — 29 | Had the upper part of his foot abraded, which was shortly afterwards attacked with erysipelas. Although the erysipelas soon disappeared, the sore, though small, healed slowly. Sleeps next to Wilson, and messes next to Smith. |
| Patrick Kane (a second attack) | June 24 | July 1 | He was this time attacked below the knee in a similar manner to Wilson and Smith. |
| Tho. Grossard | — 28 | — 9 | Was affected in the usual place below the knee. On the 2d of July the glands in the groin of the same leg, and the skin covering them, were also attacked with erysipelas. Could the disease be extended through the lymphatics? He messes next to Wilson, |

Stephen

| Men's Names. | When put on sick list. | When disch$^{d}$ sick list. | Part affected, and Remarks on the Cases. |
|---|---|---|---|
| Stephen Pitts | July 4 | July 12 | Right leg affected. He is a messmate of Grossard. |
| Peter Butler (an old man) | — 11 | — 25 | Strained his knee. A week afterwards the part injured became affected with erysipelas, which extended to the ancle, and left an œdematous swelling of the leg, tedious to remove. He sleeps next to Kane. |
| Gideon Ross | Sept. 17 | Sept. 23 | Erysipelas about the ancle. Sleeps near Grossard. He is next messmate to Wilson; he messes with Ritchy, and next to Philips and Cassey, persons afterwards affected. |
| Samuel Atkins | — 21 | — 28 | Fore-arm affected from the wrist to the elbow. He messes next to Grossard and Pitts. |
| Ja. Mathews | Oct. 1 | Oct. 7 | Left leg affected. He messes with Atkins. |
| T. Clotworthy | — 6 | — 9 | Hand affected. He also messes with Atkins. |
| Charles Lovell | — 7 | — 18 | Got a nail run in his heel, and the parts around the puncture assumed the erysipelatous inflammation. Pus was first secreted from the orifice, which in a few days changed to serum. He messes with Butler. |
| Tho. Wilson | — 20 | — 25 | A second attack. Leg affected. |
| John Smith | Nov. 14 | Nov. 22 | Erysipelas in the usual place below the knee. Messes next to Tho. Smith and Kane. |
| Michael Cassey | — 21 | — 27 | Left ancle affected. Messes next to Wilson. |
| Charles Lovell | Dec. 15 | Dec. 29 | Erysipelas returned in the heel. |
| Louis Rozer | — 26 | Jan. 14 | Hand and fore-arm so violently affected as to threaten mortification. Suppuration at length took place, situated over the back part of the metacarpal bones. It is singular that although nearly half a pint of healthy pus was discharged on opening the abscess, yet in four days after the discharge became perfectly serous. This unexpected circumstance suggested to me that the diffused, or, as it is termed, uncircumscribed tumor of erysipelas might be owing to effusion of serum in the equal and continuous extension |

of

| Men's Names. | When put on sick list. | When disch^d sick list. | Part affected, and Remarks on the Cases. |
|---|---|---|---|
| | | | of cellular membrane. This I put to the test in the case of Pitts, as stated hereafter. Messes next to John and Thomas Smith. |
| Samuel Philips | Jan. 7 | Jan. 16 | Leg affected. Next messmate to Ross and Wilson. |
| John Lucas | — 18 | — 28 | Arm affected, and although a black man, the disease was sufficiently characteristic. He is a messmate of Philips. |
| Stephen Pitts (a second attack). | — 21 | Feb. 8 | Left leg affected. Case annexed. Next messmate to Lucas. |
| Richard Allan | — 23 | Jan. 27 | Knee affected in the usual place. He is a messmate of Pitts. |
| James Ritchy | Feb. 5 | Feb. 13 | Received a cut in the palm of his hand, which was attacked with erysipelas, and extended to the fingers and back of the hand. The sore first suppurated, and latterly discharged serum. He messes with Gideon Ross. |
| Tho. Wilson (a third time affected) | — 11 | — 15 | The disease attacked the right side of the forehead, and extended over the supercilium to the eye-lids, which were so tumefied as almost to close up the eye. |

The perusal of the above would lead us to conclude that, presuming the disease to be contagious, its sphere of action is limited to a few feet. The following partial history of the case of Pitts will introduce some observations I wish to make on erysipelas abstractly, and shew the general treatment used in the present disease.

Stephen Pitts, a robust young seaman, applied, on the 20th of last January, complaining of feelings of lassitude, general debility, and constant shiverings. On examination, his pulse beat natural, his tongue was clean, and skin as usual. As the weather was stormy, I thought he sculked, and therefore did not put him on the sick list; nevertheless he had a dose of one of the neutral salts. Next morning (21st) he came with an erysipelatous inflammation extending from the knee to the ancle, occupying nearly the whole circumference of the leg. He was affected with evident febrile symptoms, and complained of some drowsiness and much head-ache. On reference to the above list, this will be found to be a second attack of the same disease, though on the other leg. He is next messmate to Lucas, who now has the erysipelas.

Sumat

Sumat Sulph. Cupri

Pulv. Ipecac. ana. gr. x. ex aqua quam primum.

Let the affected leg be slightly surrounded with a bandage kept constantly wetted with rum, and to be kept horizontal. Vespere,—vomited freely, and has had a stool since. No alteration in the leg.

Continuetur spiritus.

22d.—Leg still much inflamed, and so tumefied as to render it of one thickness from the knee downwards. More glossy in appearance, and pits on pressure. He complains of increased head-ach aggravated by a dry cough, and of some pain in the chest.

Mitte sanguinis, ℥xx.
Rep. Sal medius.
Cont. spiritus ad crurem.

23d.—Salts operated copiously; blood firm; leg still highly inflamed, and yields an œdematous feel on pressure. Determined to ascertain if the tumefaction arose from effused serum, I thrust a lancet into the part most swollen and soft, when serum tinged with the blood from the puncture followed the lancet, but yet not so freely as the previous degree of softness had led me to expect. By squeezing I could urge the fluid at pleasure, though distant from the orifice, through the puncture, leaving the part compressed wrinkled and flabby. The puncture was covered with lint.

Cont. spiritus.

26th.—The puncture made by the lancet has discharged some healthy pus for these last two days. To-day the matter is wholly serous. Erysipelas considerably abated.

Cont. spiritus.

The remaining history of this case is no farther interesting. The small sore was dilatory in healing, and discharged serum to the last. After the erysipelas had entirely faded, an œdematous thickening of the skin remained, which stimulating embrocations and a tight bandage finally removed. I am inclined to think that a blister, repeated, and applied to a different part each time, would have proved a speedier means of discharging a part, and exciting the absorbents to carry off the remainder, of the effused serum, than the stimulus and support of a tight bandage.

There are circumstances attending this disease, the cases of which I have cited, singular in themselves, and difficult to explain, unless we admit the agency of an infecting cause. 1st, The length of time the erysipelas continued in the ship. 2dly, The impressive translation of it from messmate to messmate, and from birth to birth. 3dly, The disproportionate number of erysipelatous cases in the sick list com-

 pared pared to that of any other disease. And 4thly, That cuts, bruises, or scratches should become erysipelatous, as in the cases of Ritchy, Butler, Lovell, and Kane. All these are to me inexplicable on any other supposition than that of infection. That diet could have no effect in inducing the disease is beyond a doubt, seeing the change from salt to fresh provision was frequent, and as is customary. That climate could neither produce nor influence the disease is as assuredly ascertained, seeing the complaint underwent no change whether in the summer's heat of Gibraltar, or during the intense winter's cold of a cruize in the Bay of Biscay.

Erysipelas has received the appellations of phlegmonous, bilious, œdematous, and gangrenous, according to the form it may put on. These distinctions of course are owing to other causes, than to any specific difference in these species of the disease. Where the constitution is robust, and the climate cold, it is natural to conceive that the phlegmonous erysipelas will be most frequent; where the climate is hot, or the temperament so predisposed, bilious erysipelas will be the prevailing type; and, by a parity of fact, erysipelas will assume an œdematous or gangrenous form where the constitution is debilitated, either naturally, or induced by preceding disease, or by bad air, or in short by any of those causes that depress the vigour of vital action. Permit me also to advance, *as possible*, erysipelas may take its peculiar type from the strength and nature of a contagion prevalent.

Doctrine apart, I cannot too confidently praise the eminent qualities of spirituous applications in erysipelas over all others. Farinaceous powders I suspect to be perfectly inert, and their use tolerated sheerly through the authority of prescription, and their being at least harmless. If matter should at any time exude, it can be nothing but the serum that escapes from the cellular membrane, and such also are the vesicles of erysipelas. The inordinate action of the extreme vessels excites the exhalents to pour out serum; and I am aware of no reason authorising us to conclude that this serum effused should be more acrid than that in the general circulating mass. Vesications, it is well known, are more numerous in the bilious and gangrenous, than phlegmonous erysipelas, as the greater existing debility in the former permits the easier and more external diffusion of the serum.

As erysipelas is more a disease of irritation than otherwise, it most commonly attacks the enfeebled and nervous. Irritability readily contracts the impression of contagion.

The œdema after erysipelas is invariably difficult to remove, for if the effusion is not discussed by absorption as the the erysipelas decreases, it is a sure mark of topical lymphatic debility induced by the disease, or it is consecutive of general systematic relaxation. I am apt to think that this unabsorbed effusion is the principal occasion of superinducing gangrene. Its presence is a proof of debility either local or general; and if the enveloping cellular membrane possesses not vitality strong enough to resist or remove the sedative effects of an extravasated fluid, it must of course be involved in the same putrefactive decomposition the effused serum must in time undergo.

GEORGE HUME WEATHERHEAD.

*Irish Station, Feb.* 23, 1814.

---

*For the Medical and Physical Journal.*

